# Formylated MHC Class Ib Binding Peptides Activate Both Human and Mouse Neutrophils Primarily through Formyl Peptide Receptor 1

**DOI:** 10.1371/journal.pone.0167529

**Published:** 2016-12-01

**Authors:** Malene Winther, André Holdfeldt, Michael Gabl, Ji Ming Wang, Huamei Forsman, Claes Dahlgren

**Affiliations:** 1 Department of Rheumatology and Inflammation Research, Institute of Medicine, Sahlgrenska Academy, University of Gothenburg, Gothenburg, Sweden; 2 Center for Cancer Research, Frederick National Laboratory for Cancer Research, Frederick, Maryland, United States of America; Indian Institute of Technology Delhi, INDIA

## Abstract

Two different immune recognition systems have evolved in parallel to recognize peptides starting with an N-formylated methionine, and recognition similarities/differences between these two systems have been investigated. A number of peptides earlier characterized in relation to the H2-M3 complex that presents N-formylated peptides to cytotoxic T cells, have been characterized in relation to the formyl peptide receptors expressed by phagocytic neutrophils in both men (FPRs) and mice (Fprs). FPR1/Fpr1 was identified as the preferred receptor for all fMet-containing peptides examined, but there was no direct correlation between H2-M3 binding and the neutrophil activation potencies. Similarly, there was no direct correlation between the activities induced by the different peptides in human and mouse neutrophils, respectively. The formyl group was important in both H2-M3 binding and FPR activation, but FPR2 was the preferred receptor for the non-formylated peptide. The structural requirements differed between the H2-M3 and FPR/Fpr recognition systems and these data suggest that the two recognition systems have different evolutionary traits.

## Introduction

The immune system has developed recognition systems designed to identify microbial/mitochondrial molecular patterns in the form of peptides starting with an N-formylated methionine, the first amino acid incorporated in newly synthesized proteins/peptides in prokaryotes and host cell mitochondria [1, 2]. Microbes and damaged cells/tissues release formylated peptides that are perceived as danger signals and the fMet molecular pattern is recognized by the innate immune system [3, 4]. Neutrophils, the main effector cells in our innate immune defence system express formyl peptide receptors (FPRs) that belong to the 7-transmembrane G protein-coupled receptor (GPCR) family. These receptors regulate innate immmune defense reactions and fine tune inflammatory responses [5–7]. Human neutrophils express two FPRs (FPR1 and FPR2) that share a large sequence homology and mediate fairly similar functions [8, 9]. The two neutrophil receptors bind structurally diverse agonists, most of which are receptor specific pro-inflammatory mediators that are chemotactic and activate cells to secrete granule constituents and reactive oxygen species (ROS). There are also dual agonists that activate both receptors, often with a preference for one or the other of the receptors [7]. Ligand diversity is a prominent feature of the two human neutrophil FPRs [7, 10, 11] that possess somewhat different recognition profiles. FPR1 recognizes N-formylated methionyl (N-fMet) peptides but the first FPR2 agonist described were non-formylated peptides and small molecules. Recent characterization of peptides from mitochondria and a group of phenol-soluble modulins (PSMs), from *Staphylococcus aureus*, however, clearly shows that also FPR2 has the ability to recognize N-fMet peptides but with somewhat different binding profile compared to FPR1 [3, 12–14]. It is known from earlier studies that the binding pocket in FPR1 has room for no more than five amino acids [15]. The receptor preference is, however, determined also by amino acids that do not have direct access to the orthosteric binding site [13]. The physicochemical properties as well as the size of agonistic peptides together determine both the activation potency and the receptor preference, but in order to understand the recognition mechanisms in detail, more structural information is needed.

Much less is known about the murine Fprs. It is clear that mouse neutrophils express orthologous of FPR1 and FPR2 (Fpr1 and Fpr2, respectively), but the pharmacological similarities/differences between the human and mouse neutrophil receptors have not been characterized in detail [7, 16]. It might be that different structural motifs have evolved in the recognition of formylated peptides as illustrated by the fact that the prototype high affinity FPR1 agonist fMLF is a very low affinity agonist for Fpr1. In addition to Fprs, another immune response system, the H2-M3 complex, has evolved in mice for the recognition of formylated methionine containing peptides [17]. From an evolutionary point of view, this recognition complex has developed in parallel with the Fprs. The H2-M3 complex presents N-formylated peptides from bacterial and mitochondrial metabolism to cytotoxic T cells in an MHC-class Ib restricted reaction, and this system is of particular importance for immune recognition and effector functions related to protection against intracellular bacterial pathogens such as *Listeria monocytogenesis* and *Mycobacterium tuberculosis* [18]. At the molecular level, the crystal structure of the H2-M3 reveals a non-classical binding of the formylated peptide, with a 10^4^-fold preference for binding of the N-formylated fMYFINILTL peptide over the non-formylated variant of that same peptide [19]. The presence of a formyl group is, however, not an absolute requirement since the fMet residue can be replaced by a glycine [20]. It should be noticed that the non-formylated MYFINILTL peptide used as a crystallization component in the H2-M3 complex has been suggested to be a potent agonist for the neutrophil FPR2 [21]. This suggests some similarities between the FPR- and H2-M3 recognition systems. Since no crystal structures are available for the FPRs, we set out to determine similarities/differences between the recognition systems through the functional outcome and receptor preference for H2-M3 binding peptides, using the tools availably to study FPR function.

When searching for peptides in the genome of *Mycobacterium tuberculosis*, that share features with H2-M3 binding peptides, a number of different peptides were identified [4, 22]. The abilities of these peptides to induce H2-M3-restricted cytotoxic T cell responses have been determined and even if the peptides fitted well into the M3 groove their affinities for M3 differed. The binding properties of the most potent peptides possess affinities comparable to that of another high affinity H2-M3-binding peptide derived from *Listeria* bacteria [22]. We have now determined the neutrophil activation potency of peptides earlier characterized with respect to M3-binding [19, 22], and the receptor preference for these peptides of murine and human receptors has been determined. We found all these formylated peptides to be very potent activators of neutrophils, but the structural requirements differed between the recognition systems and there were no direct correlations between the reported M3 binding affinity and the neutrophil activating capacity. The potency in receptor activation of M3^high^ peptides overlapped M3^middle^ peptides, and the peptide with the lowest M3 binding affinity was the most potent neutrophil activator. All formylated peptides activated neutrophils preferentially through FPR1 and Fpr1 in human and mouse, respectively, but with different structural requirement.

## Materials and Methods

### Materials

Percoll was obtained from Amersham Pharmacia (Uppsala, Sweden). The hexapeptides WKYMVM/m were Alta Bioscience (University of Birmingham, United Kingdom) and the phenol-soluble modulin (PSMα2, MGIIAGIIKFIKGLIEKFTGK) in its N-formylated form from American Peptide Company (Sunnyvale, CA, USA). The receptor antagonist cyclosporine H was kindly provided by Novartis Pharma (Basel, Switzerland) and PBP_10_ was obtained from Calbiochem (San Diego, USA). The formylpeptide, fMLF, the prototype agonist high affinity agonist for human FPR1 but a low affinity agonist for Fpr1, was together with isoluminol purchased from Sigma-Aldrich. The other peptides used were in one letter code fMYFINILTL (from the NADH-ubiquinone oxidoreductase chain in *Rattus norvegicus* with the protein ID P03889), its non-formylated variant MYFINILTL, and with the L-Met exchanged either for a D-Met, a non formylated Gly or fGly (fmYFINILTL; GYFINILTL; fGYFINILTL), fMILLV (from a *Mycobacterium tuberculosis* transport permease with the protent ID O33188), fMFLIDV (from a conserved hypothetical protein from *Mycobacterium tuberculosis* with the protein ID P9WF85), fMWYYLF (from a *Mycobacterium tuberculosis* acyltransferase with the protein ID O53516), fMFFLDA (from a *Mycobacterium tuberculosis* ribonuclease with the protein ID P9WF75), fMLFAAL (from a *Mycobacterium tuberculosis* permease with the protein ID P9WG17), fMIVVLV (from a *Mycobacterium tuberculosis* pyrophosphohydrolase with the protein ID P96379), fMIVIL (from a not identified protein in *Listeria monocytogenes*) and, fMIFL (from a not identified protein in *Staphylococcus aureus*), and these peptides were synthesized and high-pressure liquid chromatography-purified by Caslo Laboratory (Lyngby, Denmark) and GL Biochem (Shanghai, China). Recombinant murine tumor necrosis factor alpha (TNF-α) was from R&D Systems Europe Ltd. (Abingdon, Oxon, United Kingdom). Horseradish peroxidase (HRP) was from Boehringer Mannheim (Mannheim, Germany). All peptides were dissolved in dimethyl sulfoxide to a concentration of 10 mM and stored at –80°C until use. Further dilutions were made in Krebs-Ringer phosphate buffer that was supplemented with glucose (10 mM), Ca^2+^ (1 mM), and Mg^2+^ (1.5 mM) (KRG; pH 7.3).

### Animals

C57BL/6 wild type mice were purchased from Charles River Laboratories and Fpr2^−/−^ mice were generated as described previously [23]. Based on the fact that fMIFL and PSMα2 are the most potent selective agonist for Fpr1 and Fpr2, respectively [16, 24] these two agonists used were used as control peptides. Mice were kept under standard temperature and light conditions and fed laboratory chow and water *ad libitum*, at the Department of Rheumatology and Inflammation Research, University of Gothenburg. The Ethical Committee for Animal Experimentation, Göteborg, Sweden, approved the studies performed.

### Preparation of human neutrophils

Neutrophils were isolated from buffy coats from apparently healthy adults. The erythrocytes were removed by dextran (1%) sedimentation (1 x g). The monocytes and lymphocytes were removed by centrifugation on a Ficoll-Paque density gradient. The cells were then washed in KRG and remaining erythrocytes were removed by hypotonic lysis. The neutrophils (>90% pure) were suspended in KRG at a concentration of 1x10^7^/mL and kept on ice until use. The separation/purification protocol is described in detail in [25].

The buffy coats were obtained from the blood bank at Sahlgrenska University Hospital. Ethics approval was not needed since the buffy coats were provided anonymously and could not be traced back to a specific individual. This is in line with Swedish legislation section code 4§ 3p SFS 2003:460 (Lag om etikprövning av forskning som avser människor).

### Preparation of polymorphonuclear leukocytes from mouse bone marrow

Mice (10–16 weeks of age) were killed by cervical dislocation, the femurs and tibias were removed and freed of soft tissue attachments, and the extreme distal tip of each extremity was cut off. KRG without Ca^2+^ and Mg^2+^ (KRG-) was forced through the bone by using a 1-ml syringe with a 27-gauge needle. After dispersing cell clumps and removing the debris, the bone marrow granulocytes were isolated according to an earlier described procedure [26] with some modifications. Briefly, cell suspensions in 2 ml of KRG (−) were laid on top of a three-layer Percoll gradient (1.095, 1.085, and 1.070 g/ml). The density of each Percoll solution was verified using density marker beads. After centrifugation at 500 × g for 30 min at 4°C in a swinging bucket rotor, the lowest band (1.085/1.095 g/ml interface) was collected as the neutrophil fraction. The remaining red blood cells were eliminated by hypotonic lysis. After a final wash with KRG (−), the cells were suspended in KRG. The cell number and population of bone marrow cells were determined by flow cytometry.

### Measurement of NADPH-oxidase activity

NADPH-oxidase activity was determined using isoluminol and HRP-enhanced chemiluminescence (CL) systems that allow for the determination of superoxide production [27, 28]. The CL activity was measured in a six-channel Biolumat LB 9505 apparatus (Berthold Co., Wildbad, Germany), using disposable 4-ml polypropylene tubes with a 1 ml reaction mixture that contained 5 or 10 × 10^4^ cells/measuring sample. The tubes containing 4 U HRP and 10 μg/ml isoluminol together with the cells were equilibrated in the Biolumat for 10 min at 37°C, after which the stimulus (100 μl) was added. The light emission was recorded continuously.

Naïve bone marrow cells produce very low level of superoxide upon stimulation and to increase this production, the cells were routinely primed for one hour at RT follow by mouse recombinant TNF-α (10 ng/ml, final concentration) priming for a period of 20 min at 37°C, after which the cells were stimulated and ROS production was determined as described above.

### Data collection

Data analysis was performed using GraphPad Prism 6.0.

## Results

### The formylated peptide fMYFINILT activates neutrophils and the preferred receptor is formyl peptide receptor 1 in both man (FPR1) and mice (Fpr1)

**Fig 1:**
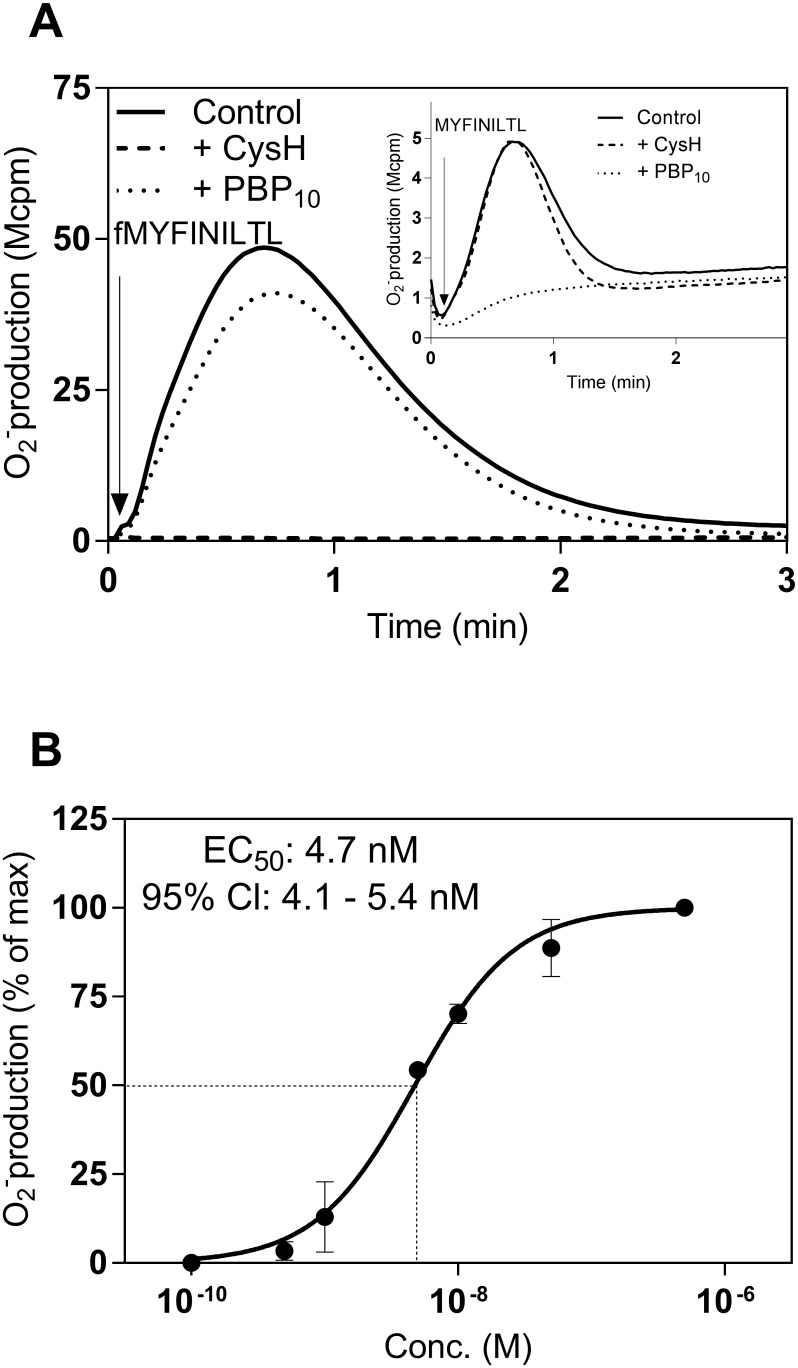
fMYFINILTL and MYFINILTL induced superoxide production from human neutrophils. Neutrophil NADPH-oxidase activity was measured by an isoluminol/HPR chemiluminescence system. (**A)** Neutrophils were pre-incubated at 37°C for 5 min in the absence (solid line) or presence of FPR1 antagonist Cyclosporin H (CysH, 1 μM, dashed line) or FPR2 antagonist PBP_10_ (1 μM, dotted line) before stimulation with fMYFINILTL (10 nM; time for addition indicated by an arrow). The curves are from one representative response (n > 10). **Inset:** The superoxide production induced by the non-formylated peptide variant MYFINILTL (1 μM) in the presence or absence (control) of FPR specific inhibitors. Abscissa, Time of study (min); ordinate, superoxide production (in arbitrary light units, cpmx10^-6^). **(B)** Neutrophil NADPH-oxidase activity induced by different concentrations of fMYFINILTL. The peak values of the responses in relation to the concentration of fMYFINILTL was determined and is expressed as a percent of the maximal response. Data are expressed as mean ± SD from four independent experiments. Abscissa, agonist concentration (M); ordinate, superoxide production (percent of max).

The human neutrophil activating effect of the peptide fMYFINILTL, earlier used as a crystallization partner to determine the structure of the formyl peptide binding site of the MHC class Ib molecule H2-M3 [19], was determined using a sensitive NADPH-oxidase assay as the read out system. This formylated peptide activated the neutrophil NADPH-oxidase with a time course of superoxide production similar to that earlier described for other FPR agonists (Fig 1A). There was a clear dose dependency of the response with an EC_50_ value in the low nM range (≈ 5 nM; Fig 1B). Based on the fact that the prototype formylpeptide fMet-Leu-Phe (fMLF), used in the same experimental set up activates neutrophils with an EC_50_ value ≈ 50 nM, we conclude that fMYFINILTL is a very potent human neutrophil activator. A very low level of activity was induced by the peptide with an identical amino acid sequence but without the formyl group at the amino terminus (Fig 1A inset). No activity was induced by MYFINILTL in neutrophils in concentrations lower than 1 μM.

Cyclosporine H and PBP_10_, two receptor specific inhibitors of FPR1- and FPR2-mediated activities, respectively [29, 30], were used to determine the receptor preference for the peptides fMYFINILTL/MYFINILTL. The activity induced by fMYFINILTL was inhibited by cyclosporine H, whereas no inhibition was seen with the FPR2 specific inhibitor PBP_10_ (Fig 1A). FPR1 was thus the preferred receptor for fMYFINILTL, and the peptide was a full agonist as the same level of activity was reached as that induced by the prototype FPR1 agonists fMLF or fMIFL. On the contrary to cyclosporine H sensitive fMYFINILTL, the low but still measurable activity induced by the non formylated peptide was inhibited by PBP_10_ but not by cyclosporine H (Fig 1A inset), suggesting that the formyl group of the peptide was of importance not only for the neutrophil activating potency but also for the FPR1/FPR2 preference.

**Fig 2:**
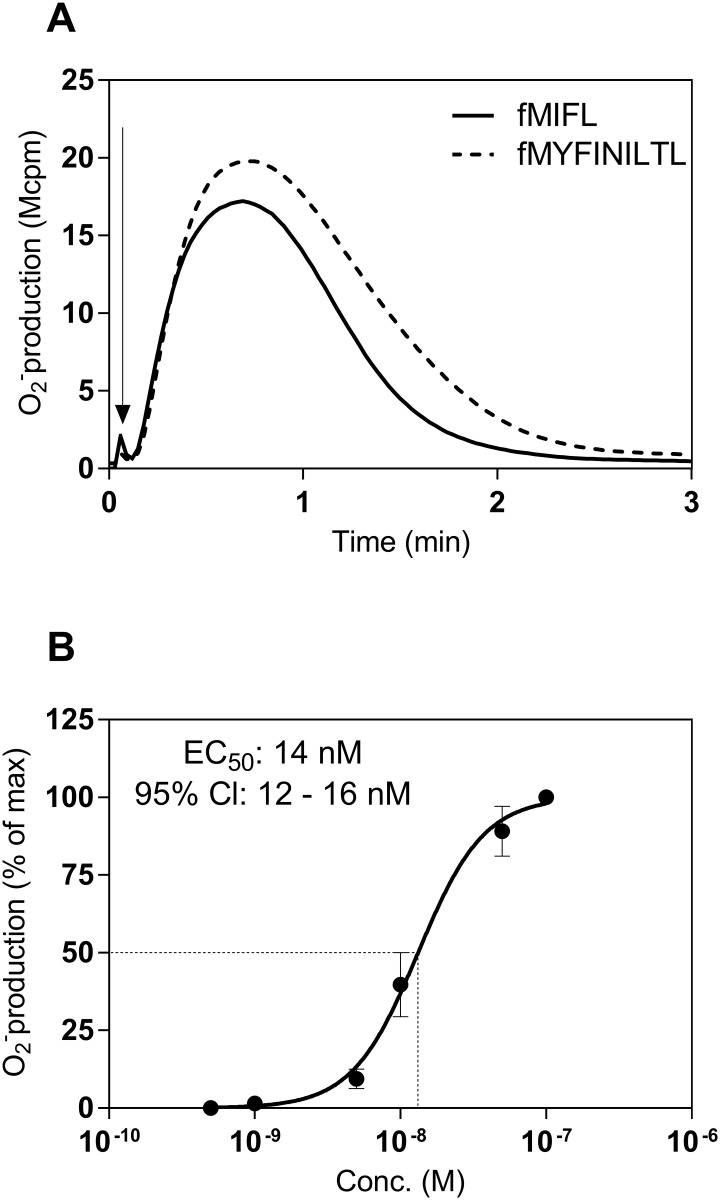
fMYFINILTL induced superoxide production from bone marrow derived mouse neutrophils. Neutrophil NADPH-oxidase activity was measured by an isoluminol/HPR chemiluminescence system. (**A)** Neutrophils (5x10^4^) were activated by either fMYFINILTL (50 nM) or fMIFL (10 nM). The time point for addition of agonists is indicated by an arrow and the curves are from one representative response (n > 3). Abscissa, Time of study (min); ordinate, superoxide production (cpmx10^-6^). **(B)** Mouse neutrophil NADPH-oxidase activity induced by different concentrations of fMYFINILTL. The peak values of the responses in relation to the concentration of fMYFINILTL was determined and is expressed as a percent of the maximal response. Data are expressed as mean ± SD from three independent experiments. Abscissa, agonist concentration (M); ordinate, superoxide production (percent of max).

The ability of the fMYFINILTL/MYFINILTL peptides to activate bone marrow derived murine neutrophils was determined using the same NADPH-oxidase assay as read out system. The formylated fMYFINILTL peptide was found to be a very potent activator also for mice neutrophils with a time course similar to that earlier described for the response induced by Fpr agonists (Fig 2A). There was a clear dose dependency of the response with an EC_50_ value also here in the low nM range (Fig 2B). No activity was induced by the MYFINILTL peptide (tested in concentrations up to 10 μM) lacking the formyl group (data not shown).

**Fig 3:**
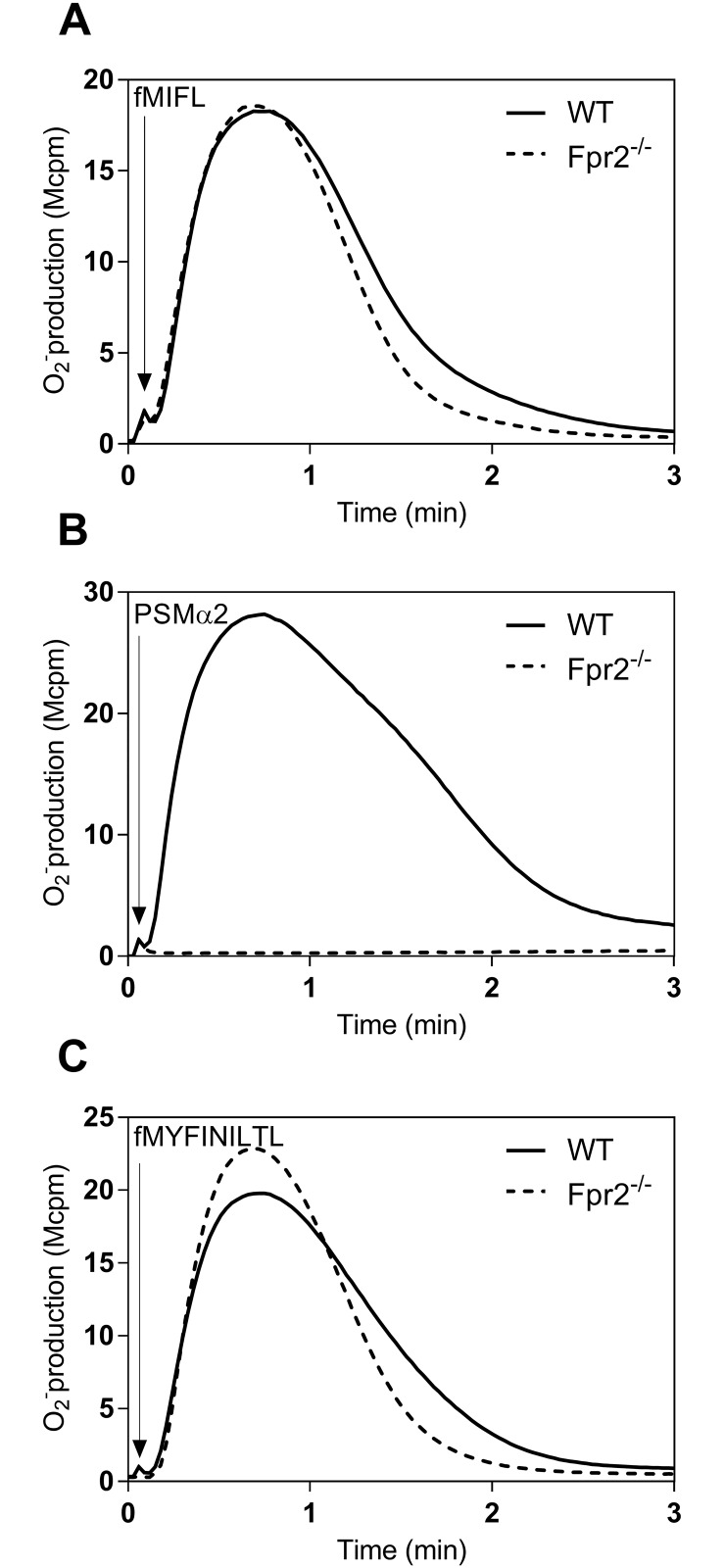
NADPH-oxidase activity induced in bone marrow neutrophils derived from wild-type and Fpr2^-/-^ mice by fMIFL and PSMα2. The oxidase activity was measured by an isoluminol/HPR chemiluminescence system. **(A)** Neutrophils (5x10^4^) from wild type (WT; solid line) and Fpr2^-/-^ (broken line) were activated by 10 nM fMIFL. **(B)** Neutrophils (5x10^4^) from wild type (WT; solid line) and Fpr2^-/-^ (broken line) were activated by 50 nM PSMα2. **(C)** Neutrophils (5x10^4^) from wild type (WT; solid line) and Fpr2^-/-^ (broken line) were activated by 50 nM fMYFINILTL. Arrows indicate the time points for agonist addition and a representative experiment out of four is shown. Abscissa, Time of study (min); ordinate, superoxide production (cpmx10^-6^).

To determine receptor preference, we used neutrophils isolated from wild type (WT) mice and from animals deficient in Fpr2 (Fpr2^-/-^), and as a proof of the concept we used the *S*. *aureus* derived formyl peptides fMIFL [16, 24] and PSMα2 [12, 14] as receptor selective agonists. There was no difference in the activity induced by fMIFL between neutrophils isolated from WT and Fpr2^-/-^ animals (Fig 3A). Neutrophils from wild type mice responded well to PSMα2, whereas no activity was induced by this peptide when Fpr2^-/-^ neutrophils were used (Fig 3B), showing that WT/Fpr2^-/-^ cells can be used as valuable tools to determine receptor preference for formylated peptides in mice. Accordingly, the receptor preference for fMYFINILTL was determined in neutrophils isolated from wild type animals expressing Fpr1 and Fpr2 and Fpr2^-/-^ animals lacking Fpr2 but expressing Fpr1. The activity induced by fMYFINILTL was comparable in WT and Fpr2^-/-^ neutrophils (Fig 3C).

Fpr1 was thus the preferred receptor for fMYFINILTL also in mice and the removal of the formyl group was critical for the ability to activate the murine receptor.

### Formylated peptides possessing different levels of M3 binding affinities activate neutrophils but with different potencies in cells from men or mice

Formyl peptides originating from *Mycobacterium tuberculosis* (*Mt*) bacteria, have earlier been shown to share features with peptides that bind the MHC class Ib protein M3 [22]. A number of such *Mt* peptides (listed in the materials and methods section) have been characterized in this study, regarding their activation profiles in relation to both human and mouse neutrophils. The *Mt* peptides have together with a control peptide from *Listeria* bacteria earlier been classified as high-, medium- and low-binders, respectively, in their ability to induce M3 expression on P338-M3 transfected cells. The relative affinity values of these peptides for M3, derived from the data presented by [22], varied from 20 (low) to 50 (high; S1 and S2 Figs).

All the *Mt* peptides were potent activating agonists measured as the NADPH-oxidase activity induced in human neutrophils, with efficacies close to that of fMIFL and they were all full agonists even if the concentration needed to induce this activity varied from around 100nM (for fMYFINILTL; fMILLV; fMLFAAL) to 1μM (for fMFLIDV). Moreover, the responses induced by the *Mt* peptides were concentration depended with EC_50_ values varying from around 100 nM to below 1 nM (S1 Fig). All the *Mt* peptides were potent activating agonists also for mouse neutrophils, with potencies close to that of fMIFL, and in mice they should similarly all be regarded as full agonists. The mouse neutrophil responses induced by the *Mt/Listeria* peptides were also concentration dependent with EC_50_ values varying from 25 nM to below 1 nM (S2 Fig). The three peptides with the highest M3 affinity (fMILLV, fMIVVLV and fMIVIL) were very potent neutrophil activators with EC_50_ values in the low nM range for both human and mouse cells, but with different relative potencies. The fMIVVLV peptide was more potent on mouse cells than on human cells whereas the order of potency was reversed for fMIVIL. The fMILLV peptide was equally potent for receptors in the two species. The activation potencies of the two peptides with M3 affinity in the middle range (fMFLIDV and fMFFLDA) were also potent activators of both human and murine neutrophils, with potencies for fMFFLDA very similar in the two species. For fMFLIDV, it was much more potent for mouse (EC_50_ ~ 1 nM) than for human receptors (EC_50_ ~ 100 nM). Of the two peptides with the lowest binding affinity for M3 (fMWYYLF and fMLFAAL) the fMWYYLF peptide was more potent for murine cells than for human cells, whereas for the fMLFAAL peptide the potencies were reversed. There was no direct correlation between the abilities of the peptides to bind M3 and the activation potencies for human or mouse neutrophils (R^2^ value > 0.05; S3A and S3B Fig) and in fact, one of the two peptides with the lowest M3 binding affinity was the most potent to activate human neutrophils (fMLFAAL) whereas the other peptide was the most potent to activate mouse neutrophils (fMWYYLF). In addition, there was no direct correlation between the potencies of the peptides to activate human neutrophils and their potencies to activate mouse neutrophils (S3C Fig). In fact one of the most potent peptides in relation to murine cells (fMFLIDV) was the least potent to activate human cells.

### All formylated peptides possessing M3 binding affinities preferred formyl peptide receptor 1 in both men and mice

**Fig 4:**
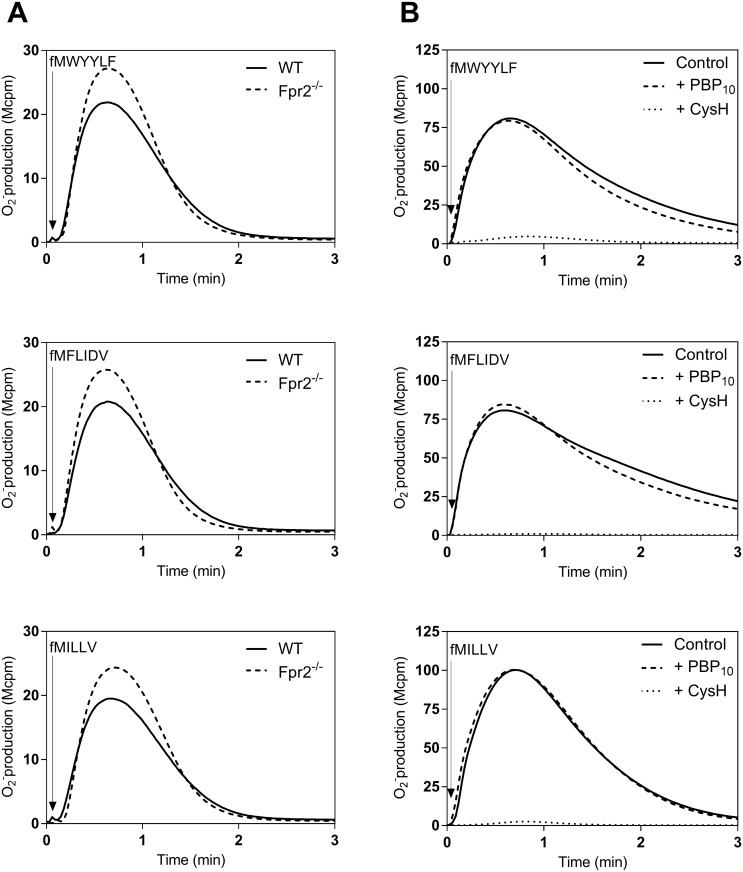
NADPH-oxidase activity induced in mouse (A) and human (B) neutrophils by formyl peptides differing in their M3 binding properties, fMWYYLF (a representative of the low M3 binding group), fMFLIDV (a representative of the middle M3 binding group) and, fMILLV (a representative of the low M3 binding group). **(A)** Neutrophils (5x10^4^) from wild type (WT; solid lines) and Fpr2^-/-^ (broken lines) were activated by by fMWYYLF (5 nM), fMFLIDV (5 nM) or fMILLV (5 nM) and the oxidase activity was measured. The time point for addition of the agonist is indicated by an arrow. **B)** Human neutrophils incubated 5 min without (solid lines) or with an FPR antagonist, either the FPR2 antagonist PBP_10_ (1 μM, dashed lines) or the FPR1 antagonist CysH (1 μM, dotted lines) were activated with fMWYYLF (100 nM), fMFLIDV (500 nM) or fMILLV (5 nM) and the oxidase activity was measured. The time point for addition of the agonist is indicated by an arrow. Arrows indicate the time points for addition of the peptide agonists. One representive experiment out of three is shown. Abscissa, Time of study (min); ordinate, superoxide production (cpmx10^-6^).

All formylated peptides induced dose-dependent activation of human neutrophils as well as of murine neutrophils, with different relative potency. In order to determine the receptor preference in human and mouse neutrophils, we used receptor selective inhibitors (cyclosporine H and PBP_10_) and neutrophils from wild type and Fpr2^-/-^ animals. The activities induced by the peptides were fairly similar in WT and Fpr2^-/-^ neutrophils demonstrating that Fpr1 was the preferred receptor for all these peptides (shown for one of the peptides in each M3 binding group in Fig 4A). In human neutrophils, the activity induced by all the peptides was inhibited by the FPR1 specific inhibitor cyclosporine H, whereas no inhibition was seen with the FPR2 specific inhibitor PBP_10_ (shown for one of the peptides in each M3 binding group in Fig 4B). FPR1/Fpr1 is thus the preferred receptor for all the peptides.

### The formylmethionyl group cannot be replaced by a glycine but the D-Met containing peptide fmYFINILT retains some FPR activating property

**Fig 5:**
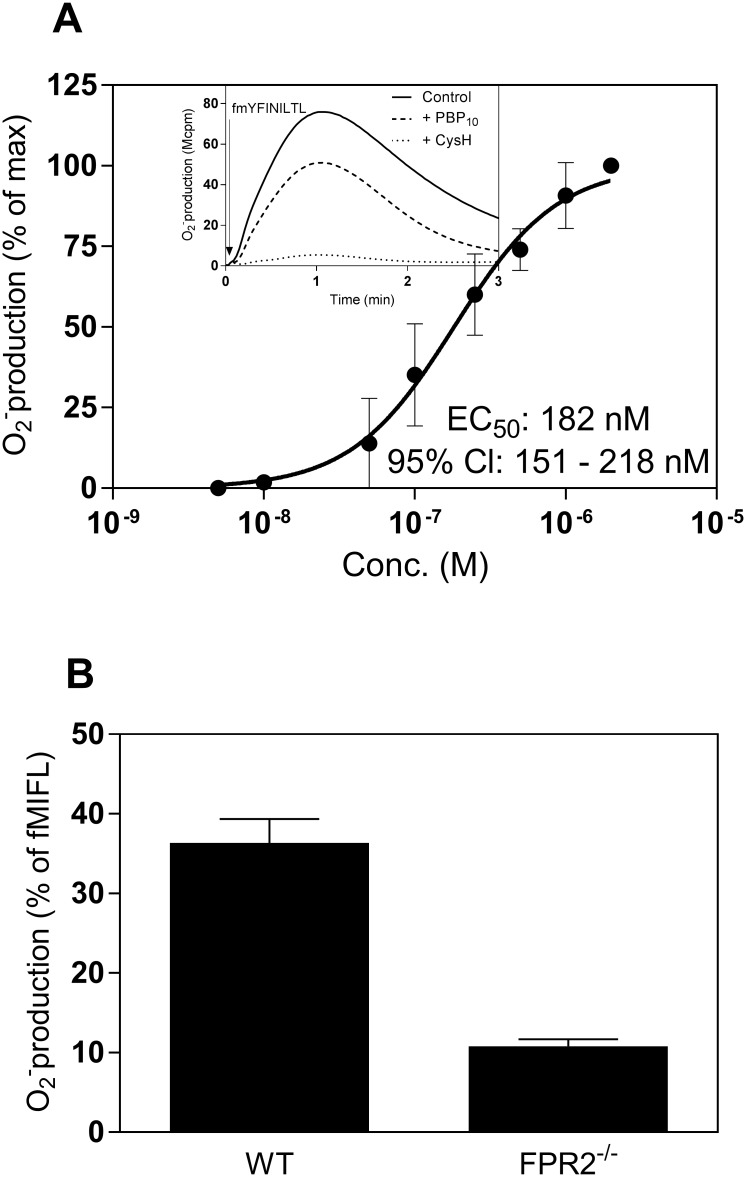
The fmYFINILTL peptide activates both human and mouse neutrophils. **A.** Dose response of the superoxide release induced by fmYFINILTL from human neutrophils. The peak values of the responses in relation to different concentrations of fmYFINILTL were determined and are expressed as percent of the maximal response. Data are expressed as mean ± SD from three independent experiments. **Inset:** Human neutrophils were pre-incubated in the absence (solid line) or presence of FPR2 antagonist PBP_10_ (1 μM, dashed line), or FPR1 antagonist CysH (1 μM, dotted line) followed by stimulation with fmYFINILTL (250 nM). An arrow indicates the time for addition of the agonist. Representive responses from one experiment out of three are shown. Abscissa, Time of study (min); ordinate, superoxide production (cpmx10^-6^). as indicated by the arrow. The figures represent one out of three experiments. **B.** The fmYFINILTL peptide (2 μM) induced superoxide production from wild type or Fpr2^-/-^ neutrophils. Data are expressed as mean ± SD of the peak response of fmYFINILTL in percent of the peak fMIFL response (n = 3).

M3 binding data with peptides containing different substitutions that replace the N-terminal f-Met reveal that a glycine can substitute the N-formyl moiety in M3 binding. In addition, the peptide interaction is stereospecific in that all M3 activity is lost when the N-terminal fMet L-isomer is replaced by the corresponding D-isomer [20]. We replaced the fMet moiety in fMYFINILTL with either a glycine (formylated and non-formylated) or a D-Met, and used these modified peptides as activators of neutrophil function. The D-isomer fmYFINILTL retained the ability to fully activate human neutrophils through FPR1 but the potency was much reduced (EC_50_ value of ≈ 200 nM compared to ≈ 5 nM for the L-isomer fMYFINILTL) (Fig 5A). The fmYFINILTL peptide was a partial agonist for mouse neutrophils with Fpr1 as a preferred receptor (Fig 5). Neither human nor mouse neutrophils could be activated by the glycine-containing peptide GYFINILTL or by its formylated variant (fGYFINILTL) when tested with concentrations up to 2 μM (data not shown).

## Discussion

The initial amino acid in bacterial as well as mitochondrial synthesis of new proteins is a methionine that contains a formyl group N-terminally. The production of formylmethionyl containing peptides/proteins is thus a unique hallmark of bacterial/mitochondrial metabolism. This molecular pattern is used by the mammalian immune system as a basis for recognition of microbial pathogens and to identify/recognize damaged tissues, and two different recognition systems have been developed to serve these functions. Accordingly, formylated peptides are recognized by a group of G-protein coupled chemoattractant receptors, the FPRs, expressed by phagocytic effector cells that constitute an important part of the innate immune system [5, 7]. These receptors mediate trafficking of phagocytes to sites of bacterial infection and/or tissue injury. N-formylmethionyl peptides are also specifically recognized by the mouse MHC class Ib antigen-presenting molecule H2-M3. This molecule binds formyl peptides and presents them to cytolytic T-lymphocytes that mediate specific clearance of primary infections as part of the adaptive immune system [4]. A number of N-formylated peptides originating from bacteria and mitochondria have been identified as ligands of H2-M3, and the binding properties of such peptides to the M3 protein have been defined through co-crystallization of the two binding partners [19]. Functional properties have also been defined through direct binding and through the outcome of this binding in relation to the abilities of peptides originating from *Mycobacterium tuberculosis* bacteria, to elicit M3-restricted T-lymphocyte cytotoxicity of infected cells [22]. We now show that formylated peptides possessing different affinities in binding to the M3-protein, potently and selectively also activate one of two FPRs expressed on neutrophils but with different structural requirement for the receptors expressed on cells of mice and men, respectively. No direct correlation was seen between the M3 binding affinity and the neutrophil activation potency.

Structural data clearly show that the M3 protein is highly dependent on the N-formylmethionyl terminus group for recognition/binding, but fairly promiscuous regarding the “down stream” structures [19]. The M3 binding peptide fMYFINILTL, originating from rat mitochondrial ND1 protein, was recognized also by neutrophils and these cells express two formyl peptide receptors (FPRs) with different recognition profiles [5, 7]. Different strategies were used to determine the receptor preference of M3 binding peptides related to activation of human and the murine neutrophil, respectively. To determine the receptor preference in human neutrophils, receptor specific inhibitors of FPR mediated activities were used as molecular tools. Cyclosporine H and PBP_10_ [29, 30], were used to determine the receptor preference in human cells. These are very specific FPR inhibitors [29, 30], but they have no or very limited effects on the mice Fpr orthologous [24]. To determine receptor preferences in mice we used a receptor knockout strategy. The difference in the activities induced by a peptide in neutrophils from wild type and from Fpr2^-/-^ animals defined the receptor preference. FPR1/Fpr1 was found to be the preferred receptor for all the formylated M3 binding peptides, suggesting some recognition similarities between M3 and the neutrophil FPR1/Fpr1. In agreement with this, presence of the formyl group, shown to be of outmost importance for M3 binding, was found to be of importance also for interaction with the FPR1/Fpr1. No activation of mice neutrophils was induced by the MYFINILTL peptide lacking the formyl group, and very high concentrations were required to activate human neutrophils with this peptide. It should also be noticed that the very large reduction in the potency was accompanied by a change in receptor preference. FPR1 was the preferred receptor for fMYFINILTL whereas FPR2 was the preferred receptor for the non-formylated variant of the peptide.

Formylated peptides from *Listeria monocytogenes* and *Mycobacterium tuberculosis* are particular interesting in vaccine development as they are presented by MHC class Ib molecules, which are generally conserved between different individuals, unlike the polymorphism of MHC class Ia molecules [31]. A group of peptides derived from *Mycobacterium tuberculosis* proteins with N-termini that share features with known M3-binding peptides and that fit to the binding groove of the M3-protein, have earlier been characterized and defined as high, modest or low affinity M3 binders [22]. In this study, we have determined activities induced by M3 binding peptides in neutrophils and show that all these peptides activate these cells but there was no direct correlation between the M3 binding characteristics and the neutrophil activation potencies. All these peptides were very potent activators of both human and murine neutrophils and the preferred receptor was FPR1/Fpr1 for them all. Out of the eight murine members of the Fpr gene family, the Fpr1 and Fpr2 are most similar to the human receptors both when it comes to sequence similarities, expression patterns, and functional properties [7]. It should be noticed that until recently no formylated peptide had been shown to activate FPR2/Fpr2, but it is now clear that PSMα peptides are specifically recognized by these receptors [12, 14, 24, 32]. We know now that FPR2 displays also binding affinity for formyl peptides, but the peptide size matters, i.e., large formyl peptides prefer FPR2 whereas short formyl peptides have high affinity for FPR1 [12, 13, 33]. The peptide size, the C-terminal composition, and charge of formyl peptides all appear to play more important roles for activation of FPR2 than of FPR1 [34]. Our data showing that none of the formylated M3 binding peptides activated neutrophils through FPR2/Fpr2, suggest that similarities (if any) should involve the M3 protein and FPR1/Fpr1, the preferred receptors for all the H2-M3 binding peptides included in this investigation. There was, however, no direct correlation between H2-M3 binding and the activity induced through activation of FPR1/Fpr1, and even if removal of the formyl group resulted in a large reduction in H2-M3 binding as well as in the neutrophil activation potency, the structural requirements differed between the two recognition systems. This is most clearly illustrated by the fact the no neutrophil activation was induced by the peptide in which the fMet residue was replaced by a glycine whereas a D-methionine containing peptide retained activity. Identical peptide modifications have diametrically opposite effects on M3 binding, implying that the FPR1/Fpr1 and M3 recognition systems have evolved through different routs.

The prototypic formyl peptide fMLF activates both FPR1 and Fpr1 but the affinity for the two receptors markedly differs [16]. This *E*.*coli* derived peptide binds to FPR1 with high affinity whereas the peptide is 1000-fold less potent for the low affinity binder Fpr1. In contrast the N-formylated peptides derived from *Listeria monocytogenes* (fMIVIL), *Staphylococcus aureus* (fMIFL) and, *Mycobacterium tuberculosis* were all very potent agonists for both FPR1 and Fpr1, and in fact some of the peptides were more potent in activating mouse cells than human cells. In accordance with the results for FPR1 there was no direct correlation between the M3 binding characteristics and the Fpr1 activation potency or between the potencies in mice and men. Even though all peptides were recognized by the same receptor, the potency varied and this was evident also between the two species as illustrated by the fact that the most potent peptide for Fpr1 was the least potent for FPR1 and inversely.

Two immune recognition systems designed to recognize bacterial/mitochondrial peptides starting with an N-formylated methionine exist, but although the formyl group in the N-terminus was of outmost importance in both recognition systems there was no correlation between the activities in the two systems. FPR1/Fpr1 was the preferred receptor for all H2-M3 binding peptides but the structural requirements differed between the recognition systems and the fact that there were no direct correlations between the reported H2-M3 binding affinity and the neutrophil activating capacity suggests that these recognition systems have different evolutionary backgrounds.

## Supporting Information

S1 Fig Dose-dependent activation of human neutrophil induced by formylated peptides. The peak values of the responses induced by different concentrations of A) fMILLV, B) fMIVIL, C) fMIVVLV, D) fMFLIDV, E) fMFFLDA, F) fMWYYLF, and G) fMLFAAL were determined. Data are expressed as percent of the maximal response, means ± SD, n = 4, and the figures represent the relative M3 binding affinity of different formyl peptides (taken from Chun et al (J.Exp.Med. 193:1213, 2001) using values of binding from experiments analyzing the capacity of the peptides to induce M3 expression in P388-M3 macrophages) and the potencies (EC_50_ values) of these peptides determined from the abilities to activate the NADPH-oxidase in human neutrophils. Abscissa, concentration (M); ordinate, superoxide production (percent of max).
(TIF)

S2 Fig Dose-dependent activation of mouse neutrophil activation induced by formylated peptides. The peak values of the responses induced by different concentrations of **A)** fMILLV, **B)** fMIVIL, **C)** fMIVVLV, **D)** fMFLIDV, **E)** fMFFLDA, **F)** fMWYYLF, and **G)** fMLFAAL were determined. Data are expressed as percent of the maximal response, means ± SD, n = 4 and the figures the potencies (EC_50_ values) of these peptides determined from the abilities to activate the NADPH-oxidase in mouse neutrophils. Abscissa, concentration (M); ordinate, superoxide production (percent of max).
(TIF)

S3 Fig Correlation analysis of the activities induced by the formylated peptides. **A)** Correlation analysis between the M3 binding affinity measured by the degree of surface M3 expression in P388-M3 transfectants (mean fluorescence intensity calculated from [22]) and human neutrophil activation potency measured by superoxide production (EC_50_ values). **B)** Correlation analysis between the M3 binding affinity and the mouse neutrophil activaiton potency (EC_50_ values). **C)** Correlation analysis of the activation potency (EC_50_ values) between mouse neutrophils and human neutrophils.
(TIF)
