# Supplementary figures and images for: Formylated MHC Class Ib Binding Peptides Activate Both Human and Mouse Neutrophils Primarily through Formyl Peptide Receptor 1
Source: PLoS One. 2016 Dec 1;11(12):e0167529. doi: 10.1371/journal.pone.0167529 (PMC5132201; doi:10.1371/journal.pone.0167529)

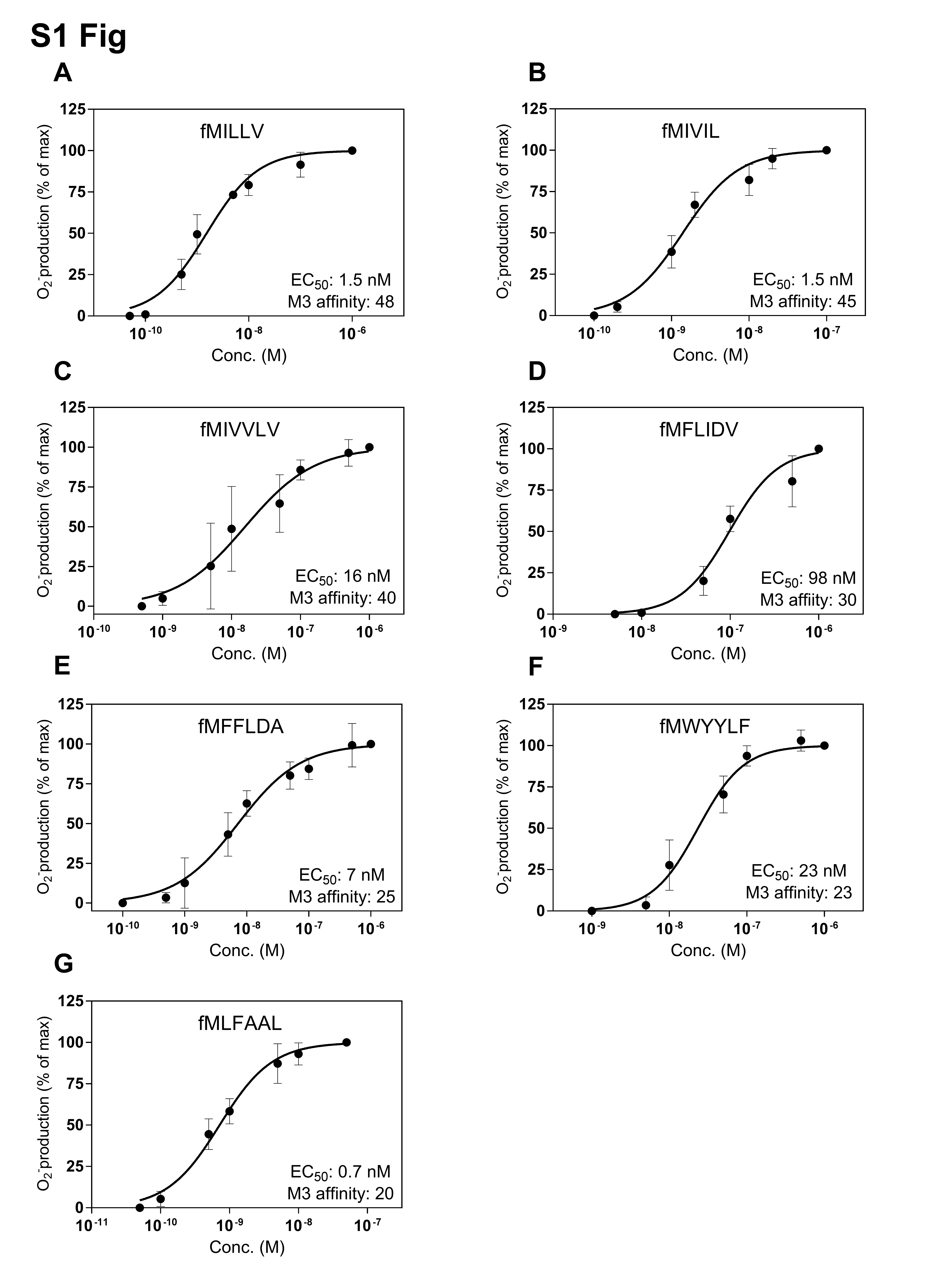

Supplement: S1 Fig — The peak values of the responses induced by different concentrations of A) fMILLV, B) fMIVIL, C) fMIVVLV, D) fMFLIDV, E) fMFFLDA, F) fMWYYLF, and G) fMLFAAL were determined. Data are expressed as percent of the maximal response, means ± SD, n = 4, and the figures represent the relative M3 binding affinity of different formyl peptides (taken from Chun et al (J.Exp.Med. 193:1213, 2001) using values of binding from experiments analyzing the capacity of the peptides to induce M3 expression in P388-M3 macrophages) and the potencies (EC50 values) of these peptides determined from the abilities to activate the NADPH-oxidase in human neutrophils. Abscissa, concentration (M); ordinate, superoxide production (percent of max). (TIF) [file pone.0167529.s001.tif]

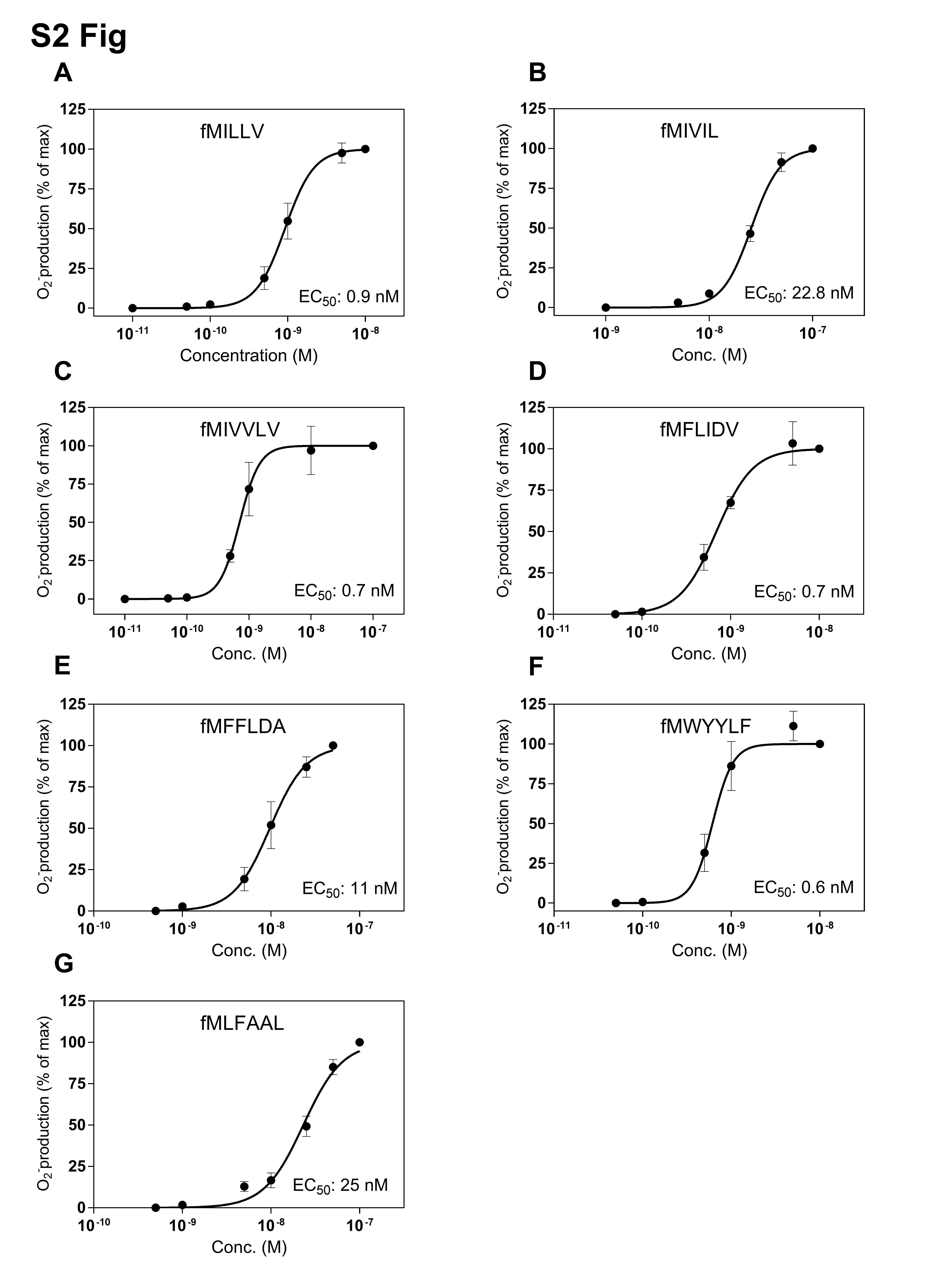

Supplement: S2 Fig — The peak values of the responses induced by different concentrations of A) fMILLV, B) fMIVIL, C) fMIVVLV, D) fMFLIDV, E) fMFFLDA, F) fMWYYLF, and G) fMLFAAL were determined. Data are expressed as percent of the maximal response, means ± SD, n = 4 and the figures the potencies (EC50 values) of these peptides determined from the abilities to activate the NADPH-oxidase in mouse neutrophils. Abscissa, concentration (M); ordinate, superoxide production (percent of max). (TIF) [file pone.0167529.s002.tif]

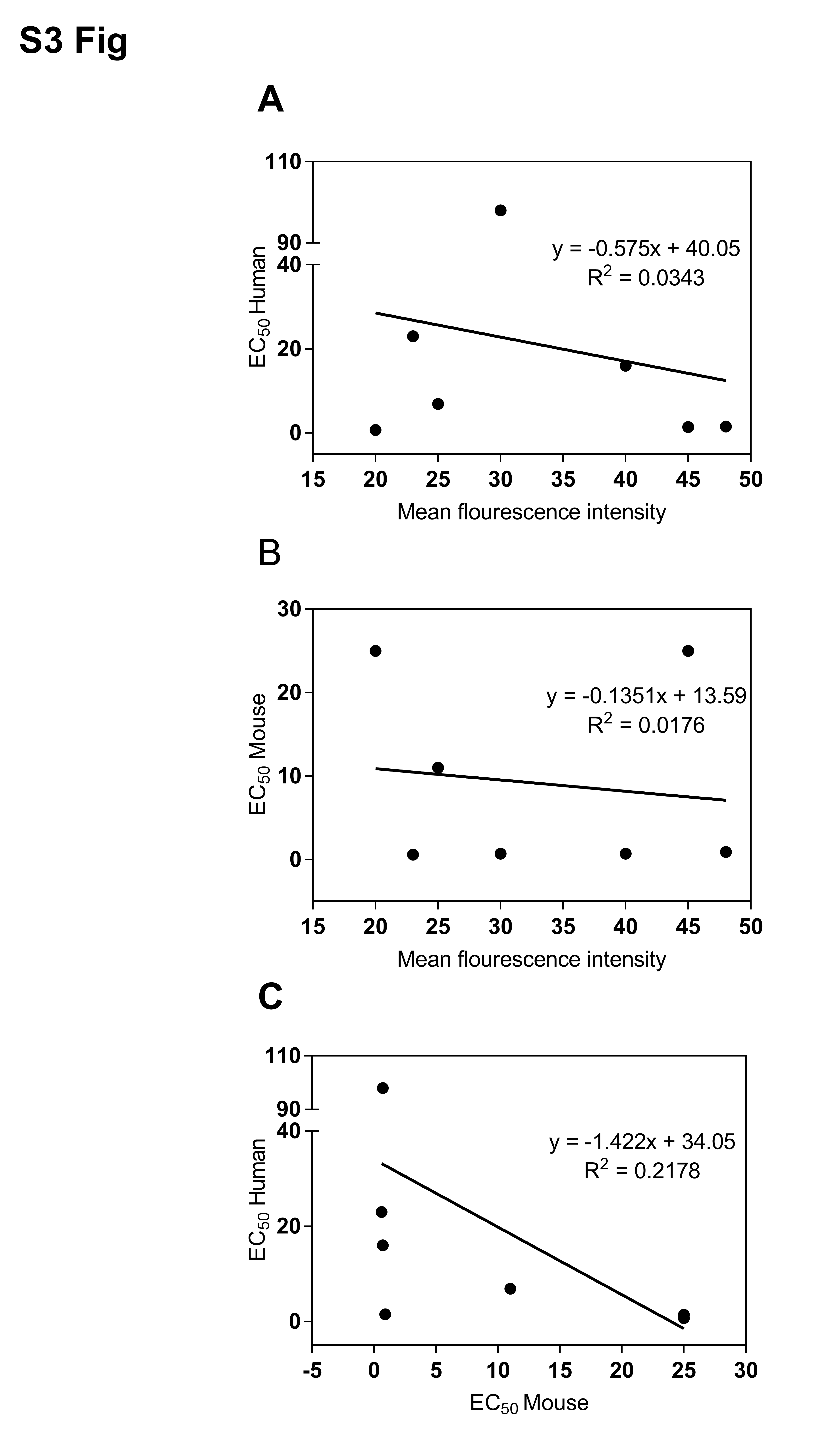

Supplement: S3 Fig — A) Correlation analysis between the M3 binding affinity measured by the degree of surface M3 expression in P388-M3 transfectants (mean fluorescence intensity calculated from [22]) and human neutrophil activation potency measured by superoxide production (EC50 values). B) Correlation analysis between the M3 binding affinity and the mouse neutrophil activaiton potency (EC50 values). C) Correlation analysis of the activation potency (EC50 values) between mouse neutrophils and human neutrophils. (TIF) [file pone.0167529.s003.tif]
